# Tuning Electrochemical Reactions with Ratchet-Based Ion Pumps

**DOI:** 10.1021/acsaem.5c02349

**Published:** 2025-10-04

**Authors:** Dafna Amichay, Alon Herman, Keren Shushan Alshochat, Eden Grossman, Baruch Hirsch, Anchal Vashishtha, Eran Edri, Brian A. Rosen, Gideon Segev

**Affiliations:** † School of Electrical and Computer Engineering, 26745 Tel Aviv University, Tel Aviv 6139001, Israel; ‡ Department of Chemical Engineering, 26732 Ben-Gurion University of the Negev, Be'er-Sheva 8410501, Israel; § Ilse Katz Institute for Nanoscale Science and Technology, Be'er-Sheva 8410501, Israel; ∥ Department of Materials Science and Engineering, 26745 Tel Aviv University, Tel Aviv 6997801, Israel

**Keywords:** ion pumps, ratchets, water splitting, electrolysis, hydrogen evolution reaction

## Abstract

Electrochemical reactions are highly sensitive to the physical and chemical environments near the electrodes. Thus, controlling the electrolyte ionic composition and the electrochemical potential of specific ions can modify the overpotential of electrochemical reactions and enhance their selectivity toward the desired products. Ratchet-based ion pumps (RBIPs) are membrane-like devices that utilize temporal potential modulation to drive a net ionic flux with no associated electrochemical reactions. RBIPs were fabricated by coating the surfaces of nanoporous alumina wafers with metals, forming nanoporous capacitors. Placing the RBIP between two electrolyte compartments and applying an alternating signal between the metal layers resulted in a voltage buildup across the membrane, leading to ion pumping. Here, we demonstrate that by modifying the electrochemical potential of ions, RBIPs can accelerate or inhibit electrochemical reactions on the surface of adjacent water-splitting electrodes according to the RBIP input signal. Proton pumping toward a water-splitting cathode prevented proton depletion due to the hydrogen evolution reaction and maintained the pH in the cathode compartment. The combination of ion pumping and ion selectivity can enable the electrolyte composition to be tuned near the electrodes, providing greater control over the electrochemical process.

## Graphical abstract

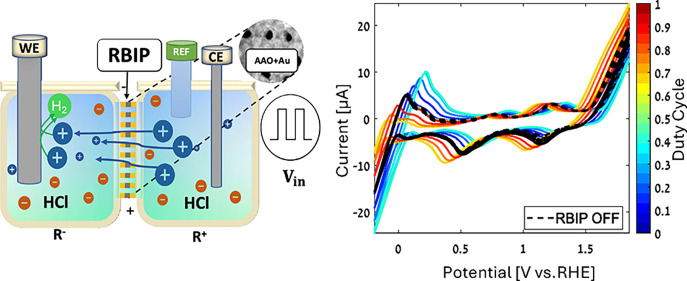

## 1 Introduction

Electrochemical reactions are sensitive to the ionic environment in the vicinity of the electrode. Thus, controlling the local ionic environment near electrodes can tune the reaction overpotential and current.^1–7^ For example, increasing the proton concentration near a cathode driving the hydrogen evolution reaction (HER) can lower the reaction overpotential and enable higher currents and efficiency.^6–9^ Furthermore, local control of the proton concentration near the cathode allows the HER to be driven without compromising the reaction kinetics while allowing a higher pH of the solution in other parts of the system. Alternatively, decreasing the proton concentration near the cathode can inhibit the HER in reactions in which hydrogen poisoning is to be avoided.^10–14^ In multiproduct reactions, pumping specific ions away from the working electrode can remove unwanted intermediate species, hinder a competing reaction, and provide another handle for controlling the reaction selectivity. For example, pumping protons away or toward a CO_2_ reduction cathode can help tune the H_2_ to CO ratio in CO_2_ reduction systems.^15,16^

Ratchet-based ion pumps (RBIPs) utilize the temporal modulation of the electric potential to drive a nonzero time-averaged ionic current with no associated electrochemical reactions.^17^ The RBIPs are fabricated by coating the two surfaces of nanoporous anodized aluminum oxide (AAO) wafers with thin metal layers, forming nanoporous capacitor-like structures. When placed as a membrane between two electrolyte compartments, the nonlinear capacitance of the electrode double layers results in a dispersion of the charging and discharging time constants at each RBIP surface. This leads to a buildup of an electric potential difference across the RBIP membrane and to a net ion flux through the RBIP. In the first experimental demonstration, the RBIP induced ionic currents on the order of 10 μA cm^–2^ and a voltage of about 80 mV. The RBIP also showed a noticeable output for signals with amplitudes as low as 50 mV (peak-to-peak), indicating that ion pumping is not carried out by redox reactions. Moreover, RBIP-driven electrodialysis was demonstrated, reaching a 50% decrease in conductivity in a dilution cell.^17^ Theoretical studies have shown that RBIPs can drive selective ion separation by transporting ions with the same charge in opposite directions according to their diffusion coefficients, or drive ambipolar transport in which both cations and anions are transported in the same direction.^18,19^ In this work, we demonstrate how RBIPs can tune the overpotential and current of electrochemical reactions by pumping protons toward or away from water-splitting electrodes. By directing protons toward a Pt cathode, the RBIP enhanced the HER and compensated for the proton depletion that resulted from the reaction. Alternatively, by pumping ions away from the water-splitting cathode, the RBIP enhanced proton depletion and increased the pH in the cathode compartment. The introduction of selective ion pumping membranes into electrochemical systems can enable the control of the overpotential of electrochemical reactions and fine-tune more complex reactions, thereby providing an additional degree of freedom for the electrochemical process.

## 2 Experimental Section

### 2.1 Sample Fabrication

AAO wafers (60 nm pore diameter and 50 μm thickness, InRedox LLC) were annealed at 650 °C for 10 h.^20^ Then, a 40–50 nm thick (planar equivalent) gold thin film was deposited on each surface by using magnetron sputtering. Last, both surfaces were coated with an 8 nm layer of TiO_2_ or Al_2_O_3_ using Atomic Layer Deposition (ALD). The TIO_2_ ALD process was as described by Vega et al.^21^ The exposure time to the precursors was set to 1 s, and the purging time was set to 5 s. ALD of Al_2_O_3_ was carried out using a Gemstar XTTM tabletop ALD system. The chamber pressure was approximately 170 mTorr, and trimethyl aluminum (TMA) and H_2_O were used as precursors. The process comprised alternating pulses of TMA and H_2_O, with the pulse lengths of 250 and 150 ms, respectively. The expo value was opened for 60 s after each pulse, and after that, Ar gas at a flow rate of 10 SCCM was purged to remove unreacted precursors from the chamber. To achieve a thickness of 8–10 nm, 60 deposition cycles were done.^22,23^ The AAO wafers were chosen for their relatively easy handling, chemical stability, and well-defined geometry. A detailed discussion on the device fabrication, stability, and the performance of wafers with various pore diameters can be found in our previous work.^17^

### 2.2 Experiment Design

All experiments were conducted in a two-compartment PEEK electrochemical cell, as shown in Figure 1a. The RBIP was placed as an active membrane separating the two compartments. Figure 1b shows an illustration of the experimental setup. A Pt working electrode with a 1.6 mm diameter working area (ALS Japan 002313, denoted as WE) was placed in one electrolyte compartment. A Pt wire counter electrode (ALS Japan no. 002233, denoted CE) and a reference electrode (Ag/AgCl in saturated NaCl, ALS Japan no. 013393, denoted RE) were placed in the other compartment. The electrode potential or current was controlled by using a potentiostat (Zhaner ZENNIUM X). The RBIP was connected to a signal generator, which provided the ratchet input signal (Keysight 33500B). In pH regulation experiments, pH measurements were taken using a Mettler Toledo micronano pH electrode. Every 30 min, three separate pH measurements were taken and averaged. The solution was refreshed after each experiment. When changing samples, the cell was cleaned using the following process: the PEEK parts were sonicated in IPA for 15 min, followed by a sonication in distilled water. Next, the cell was immersed in HNO_3_ (20%) for 2 h. Finally, the cell was sonicated in distilled water for 15 min, 3 times. The working electrode was cleaned using a commercial polishing kit (ALS Japan), alumina polishing paper, and an alumina slurry solution. It was then sonicated in distilled water for 5 min.

**Figure 1:**
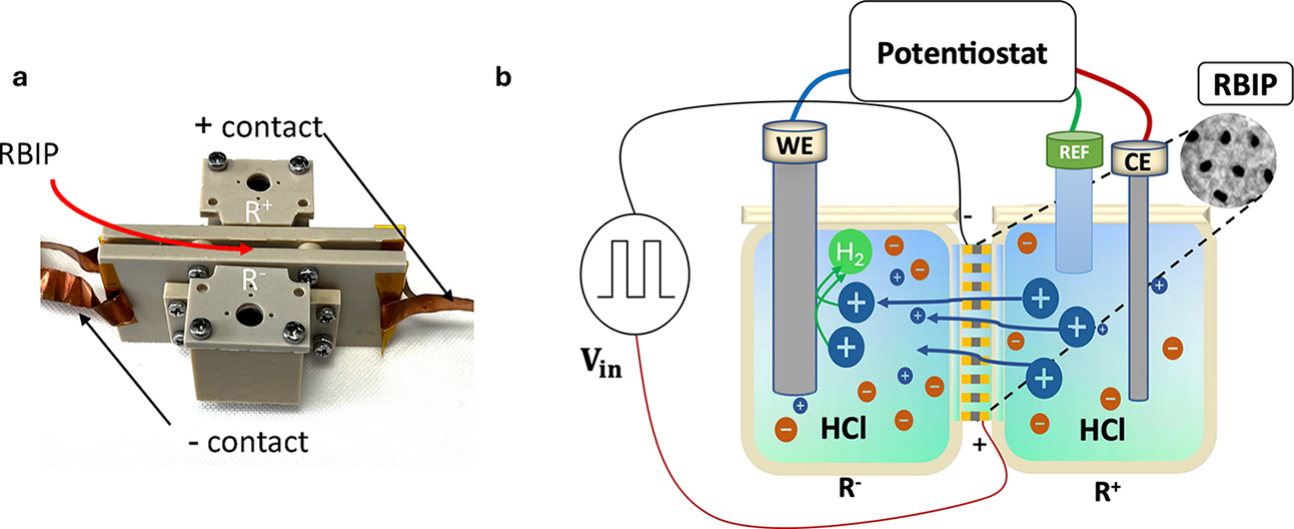
(a) Photograph of the electrochemical cell used for RBIP characterization. The RBIP is placed between the two compartments, and the copper tape is used to contact the RBIP. (b) Schematic illustration of the experimental setup. The RBIP is placed as a membrane separating the two compartments of an electrochemical cell. A signal generator connected to the RBIP provides the ratchet input signal *V*_in_. The working, reference, and counter electrodes (WE, REF, and CE, respectively) are connected to a potentiostat.

### 2.3 RBIP Performance Characterization

The RBIP performance was characterized in response to periodic square wave input signals:

$$V_{\mathrm{in}}(t)=\{\begin{array}{ll} V_{p-p}/2, & 0<t<=d_{c}T\\ -V_{p-p}/2, & d_{c}T<t<=T \end{array}$$ 1

where *V*_p–p_ is the input signal amplitude (peak-to-peak), *T* is the input signal temporal period, and *d*_C_ is the input signal duty cycle—the ratio between the time the input signal is at its high value and its temporal period. To characterize the RBIP performance, the working electrode current or potential was measured in response to the RBIP input signal. These measurements are termed duty cycle sweeps and they can be conducted in chronoamperometry mode to measure the temporally averaged RBIP-induced current, *I̅*_out_, or chronopotentiometry mode to measure the RBIP-induced voltage, *V̅*_out_. In chronoamperometry duty cycle sweeps, the working electrode (WE) potential is fixed and its current is measured while varying the input signal duty cycle. In chronopotentiometry duty cycle sweeps, the working electrode current is fixed, and the WE potential is measured while the input signal duty cycle is varied. For each duty cycle, a square wave input signal was applied to the RBIP for *t*_on_ = 30 s and then *V*_in_ was set to 0 V for *t*_off_ = 30 s. The temporally averaged RBIP-induced current (voltage) is as follows:

$${\overset{-}{I}}_{\mathrm{out}}={\overset{-}{I}}_{\mathrm{ON}}‐{\overset{-}{I}}_{\mathrm{OFF}}$$ 2

where *I̅*_ON_ is the temporally averaged WE current (potential) measured when the RBIP is ON, and *I̅*_OFF_ is the temporally averaged WE current (potential) when the RBIP is OFF:

$${\overset{-}{I}}_{\mathrm{ON}}=\frac{1}{t_{\mathrm{AV}}}\int_{(t_{\mathrm{ON}}-t_{\mathrm{AV}})/2}^{(t_{\mathrm{ON}}+t_{\mathrm{AV}})/2}I_{\mathrm{ON}}(t)dt$$ 3

$${\overset{-}{I}}_{\mathrm{OFF}}=\frac{1}{t_{\mathrm{AV}}}\int_{(t_{\mathrm{OFF}}-t_{\mathrm{AV}})/2}^{(t_{\mathrm{OFF}}+t_{\mathrm{AV}})/2}I_{\mathrm{OFF}}(t)dt$$ 4

*I*_ON_ is the current measured when the RBIP was ON, *I*_OFF_ is the current measured when the RBIP was OFF, and *t*_AV_ = 20 s is the length of the temporal window in which the output is averaged. The uncertainty of the RBIP-induced current (voltage) follows:

$$\sigma_{\overset{-}{I}}=\sqrt{{\left(\frac{\sigma_{\mathrm{ON}}}{\sqrt{n_{\mathrm{ON}}}}\right)}^{2}+{\left(\frac{\sigma_{\mathrm{OFF}}}{\sqrt{n_{\mathrm{OFF}}}}\right)}^{2}}$$ 5

where σ_ON_ and σ_OFF_ are, respectively, the standard deviations of the measurements taken within the ON and OFF averaging intervals. *n*_ON_ and *n*_OFF_ are, respectively, the number of measured data points in the ON and OFF averaging intervals.

## 3 Results and Discussion

### 3.1 Electrochemical Characterization

RBIP samples were fabricated by coating both sides of AAO nanoporous wafers (pore diameter of 60 nm) with 50 nm thick (planar equivalent) gold thin films, followed by an 8 nm thick TiO_2_ or Al_2_O_3_ layer deposited with ALD. For more details on the fabrication process, refer to the experimental section. The RBIP was placed as an active membrane between two compartments of an electrochemical cell. A platinum working electrode was placed in one compartment, and a counter electrode and a reference electrode were placed in the opposite compartment. The working, reference, and counter electrodes were connected to a potentiostat, and the two RBIP metal surfaces were connected to a signal generator. Figure 1a shows a photograph of the electrochemical cell used for the measurements, and Figure 1b shows a schematic illustration of the experimental setup. The *R* ^+^ compartment in the electrochemical cell is the compartment adjacent to the RBIP surface connected to the positive lead of the signal generator, and the *R* ^–^ compartment is the compartment adjacent to the RBIP surface connected to the negative lead of the signal generator.

To ensure that the system is chemically clean and stable, cyclic voltammetry (CV) measurements of the platinum working electrode were conducted. The CV measurements were performed in HCl (pH = 4.2, 0.2 mM and pH = 2.56, 2.75 mM), KCl (pH = 6.2, 0.2 mM), and H_2_SO_4_ (pH = 2.5, 1.6 mM) aqueous solutions. All measurements were taken in a PEEK electrochemical cell with a 3-electrodes setup (Figure 1a,b) at a scan rate of 50 mV s^–1^. More details about the setup can be found in the experimental section. Figure 2 shows the measured voltammograms. Chemical processes were assigned to each peak by comparing the CV curves to those of well-studied Pt electrodes in H_2_SO_4_ and to HCl aqueous solutions.^24,25^ The CV peaks of HCl at pH 2.56 closely align with the H_2_SO_4_ peaks, and the electrochemical water windows of both voltammograms are similar. Hydrogen under-potential deposition (HUPD) occurs when protons are absorbed to the cathode at potentials that are more positive than the equilibrium reaction voltage.^24,26–29^ The arrows in Figure 2 point to the HUPD peaks. The CV curves measured with HCl pH 4.2 and KCl show HUPD peaks that are less defined than the peaks in H_2_SO_4_ or HCl at a pH of 2.56. The HUPD peaks in pH = 4.2 HCl and KCl are indicated by dashed arrows.

**Figure 2:**
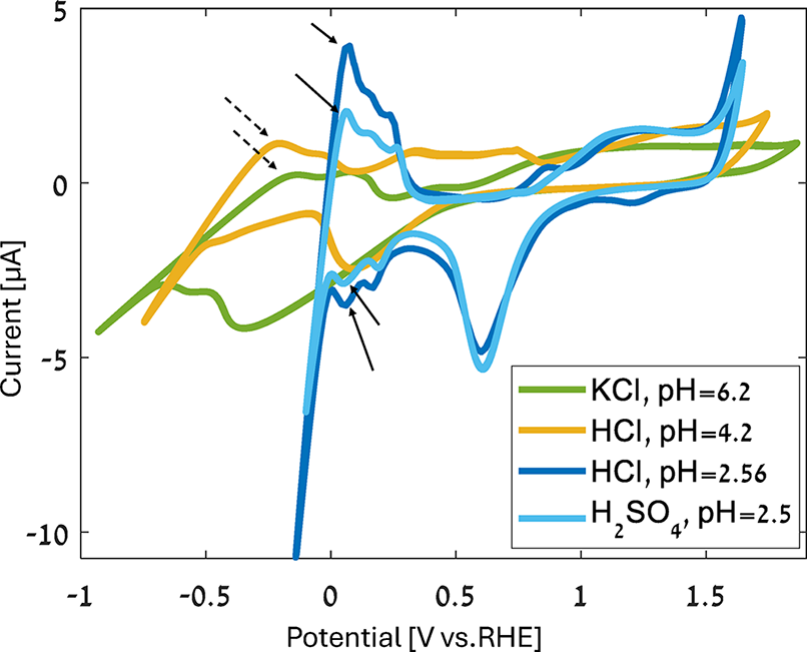
CV measurements of the platinum working electrode. The scan rate is 50 mV s^–1^, and the solutions are aqueous HCl (pH = 4.2, 0.2 mM and pH = 2.56, 2.75 mM), KCl (pH = 6.2, 0.2 mM), and H_2_SO_4_ (pH = 2.5, 1.6 mM). The arrows point to the HUPD peaks in each solution.

### 3.2 Current Enhancement

The performance of the RBIP was assessed by conducting a chronoamperometry duty cycle sweep in which the Pt working electrode (WE) potential was set to 0 V vs RHE, and the WE current was measured. The sample was fabricated as described in the experimental section with a TiO_2_ ALD coating. The electrolyte was a 0.2 mM HCl aqueous solution, the working electrode was placed in the *R* ^–^ compartment, and the counter and reference electrodes were placed in the *R* ^+^ compartment (this electrode configuration is denoted hereafter as configuration A). The input signal was set to 0 V for 30 s (RBIP OFF), after which a square wave input signal was applied for 30 s (RBIP ON). The input signal frequency was 100 Hz, and the amplitude was *V*_p–p_ = 1.4 V. The input signal amplitude was chosen to maximize the RBIP output without driving electrochemical reactions on the RBIP surfaces. The optimal frequency is determined by the time constants for charging and discharging the double layers at RBIP metal surfaces. A thorough study of the RBIP driving mechanism and response to various input signals can be found in our previous work.^17^ Figure 3a shows the current measured during the duty cycle sweep. The shaded regions mark the times when the RBIP was off, and the white regions indicate the times when a square wave input signal was applied. The color bar indicates the duty cycle of the input signal when the RBIP was ON. Figure 3c shows the current measured during a chronoamperometry duty cycle sweep in which the WE was in the *R* ^+^ compartment and the reference and counter electrodes are in the *R* ^–^ compartment (see the illustration in the inset of Figure 3c. This configuration is denoted as configuration B). The RBIP-induced current is the time-averaged current measured when the input signal is applied to the RBIP, reduced by the time-averaged current measured while the RBIP was OFF (more details on the calculation of the RBIP-induced current can be found in the experimental section). Figure 3b,d shows the RBIP-induced current as a function of the duty cycle when the system was in configurations A and B, respectively. During the HER, protons are directed toward the cathode. However, the RBIP action alters this proton flux, accelerating or hindering it, depending on the cathode’s position (configuration A or B) and the characteristics of the input signal. When a constant bias is applied (i.e., a duty cycle of 0 or 1), the RBIP-induced current is negligible and diminishes rapidly. At low duty cycles (*d*_C_ < 0.5), the RBIP drives a more cathodic current with an increase of up to 1 μA in configuration A and a less cathodic current in configuration B. Conversely, at high duty cycles (*d*_C_ > 0.5), the RBIP drives a more cathodic current in configuration B and a less cathodic current in configuration A. Thus, for *d*_C_ < 0.5, the RBIP drives protons toward the *R* ^–^ compartment, and for *d*_C_ > 0.5, the RBIP drives protons toward the *R* ^+^ compartment. Hence, at each duty cycle, the RBIP exerts a force on the protons in a direction independent of the flux induced by the electrochemical reactions at the working and counter electrodes. The anodic currents in configuration A at duty cycles above 0.5 (Figure 3a) are a result of the RBIP inducing a voltage of several dozens of mVs, which shifted the working electrode operating point to the potential of the HUPD anodic peak. In prior experimental demonstrations of RBIPs, the sign of the output did not change with the duty cycle.^17^ However, in Figure 3b,d, the RBIP output is approximately antisymmetric with respect to a duty cycle of 0.5, indicating that in this sample, the two surfaces showed a similar nonlinear capacitance.^17^ This may be a result of a change in charge distribution within the pore and in the resting potential of the electrodes in this specific solution and concentration. Engineering spatially asymmetric devices will further increase their output and determine the ion pumping direction.^17^ Nevertheless, the ratchet effect on the electrochemical reaction is always consistent: pumping protons toward the cathode reduces the overpotential and increases the current.

**Figure 3:**
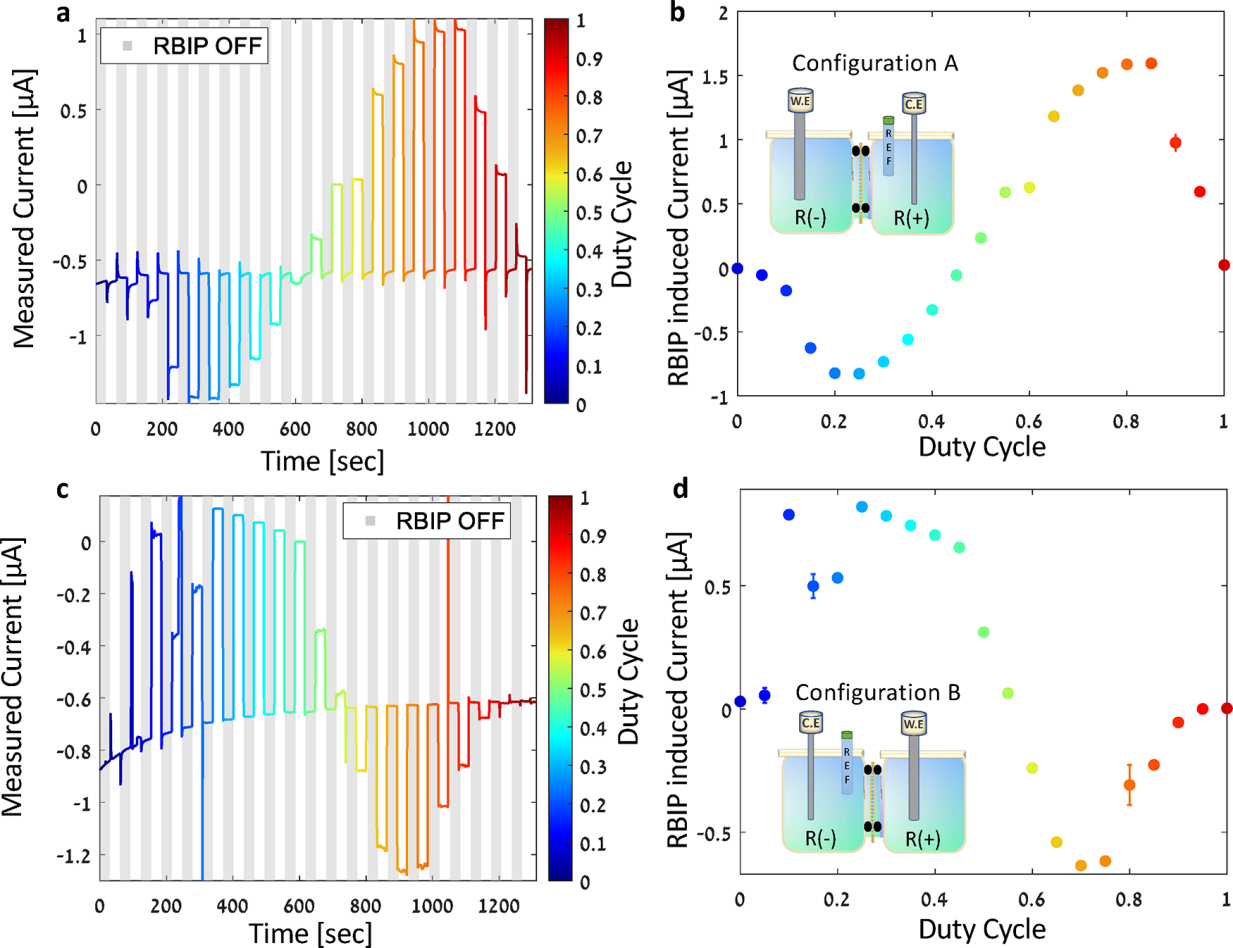
(a, c) Measured current for a system in configurations A and B, respectively. The shaded areas in a and c indicate the times when the RBIP is OFF (*V*_in_ = 0 V) and the bright areas indicate the times when it is on. The color bar indicates the duty cycle of the input signal when the RBIP is ON. The duration of each ON and OFF cycle was 30 s. (b, d) Temporally averaged RBIP-induced current as a function of the duty cycle obtained from (a, c). In all measurements, the WE potential is 0 V vs RHE. The input signal frequency is 100 Hz, and the amplitude is *V*_p–p_ = 1.4 V. The sample was fabricated as described in the experimental section with a TiO_2_ ALD coating. The compartments are filled with a 0.2 mM HCl aqueous solution. The insets in parts b and d are illustrations of configurations A and B, respectively. The error bars in (b,d) mark the uncertainty as calculated using eq 5.

### 3.3 pH Regulation

Next, we show how RBIPs can help regulate pH in the cathode compartment. To do so, the system was operated in configuration A at a constant working electrode current of −3 μA. The pH of both electrolyte compartments was measured during operation. First, a baseline measurement was performed with the RBIP disconnected. The RBIP was then operated with a duty cycle of 0.2, a frequency of 100 Hz, and an amplitude of *V*_p–p_ = 1.4 V. Lastly, the RBIP was operated with a duty cycle of 0.6 and the same frequency and amplitude. The sample was fabricated as described in the experimental section with a TiO_2_ ALD coating. Figure 4 shows the pH values of the two compartments measured in the three experiments. The RBIP pore walls are positively charged, leading to a partial permselectivity that impedes proton transport through the RBIP.^30,31^ When HER was driven at a current of −3 μA, proton consumption by the cathode was faster than proton transport through the RBIP. As a result, the proton concentration in the cathode compartment was reduced and the pH increased. Driven with a duty cycle of 0.2, the RBIP pumped protons toward the cathode. The augmented proton flux compensated for the proton consumption by the electrochemical reaction and maintained a more moderate pH in the cathode compartment. A schematic representation of the proton replenishment process is illustrated in Figure 4b. However, at a duty cycle of 0.6, the RBIP pumped protons away from the cathode compartment, increasing proton depletion and resulting in a pH higher than the baseline. This proton depletion process is illustrated in Figure 4c. The change in pH in the anode compartment is within error of the measurement system. The change in pH in response to the ratchet action demonstrates that RBIPs can regulate the electrolyte composition and pH in electrochemical systems. Repeating this experiment with a different sample and testing both electrode configurations A and B again demonstrate the directionality of the RBIP, as shown in the Supplementary Figure S1. The direction of ion pumping determines whether the RBIP enhances or mitigates proton depletion in the cathode compartment (Supporting Information, Section 1). In electrochemical systems with multiple competing reactions, RBIP can optimize the reaction selectivity. For instance, extracting protons from the cathode compartment can minimize hydrogen generation in CO_2_ reduction systems, where HER is a competitor.^15,32^ Ion pumping, as demonstrated above, can pave the way toward local pH control. For example, if operating with a small distance between the RBIP and the cathode, the RBIP can increase the proton concentration locally near the cathode, thus facilitating the HER without compromising reaction kinetics while maintaining a more moderate bulk electrolyte pH.

**Figure 4:**
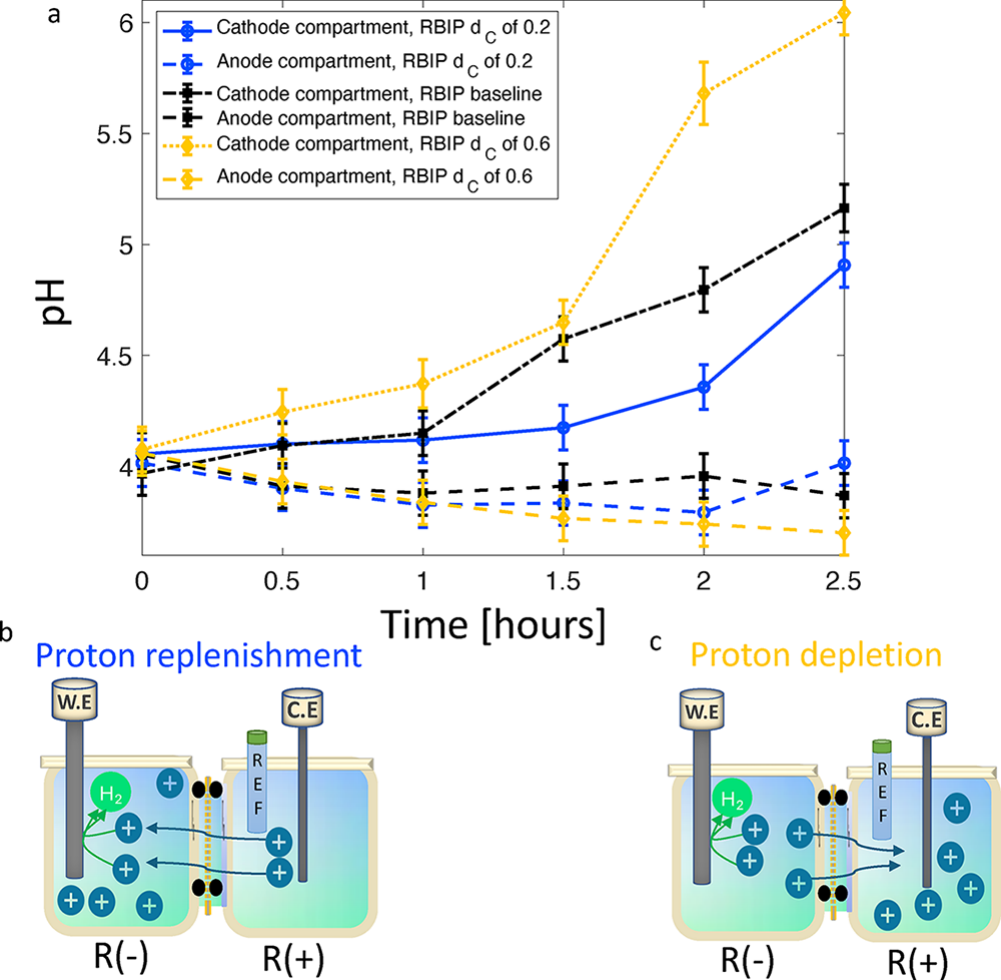
(a) pH of the anode and cathode compartments during 2.5 h of operation with the RBIP operating and when it is disconnected. The cathode current is −3 μA, and the system is in configuration A. The sample was fabricated as described in the experimental section with a TiO_2_ ALD coating. The compartments are filled with a 0.2 mM HCl aqueous solution, the input signal frequency is 100 Hz, and the amplitude is *V*_p–p_ = 1.4 V. (b) Schematic illustration of the proton replenishment process at a duty cycle of 0.2, which maintains a moderate pH in the cathode compartment. (c) Schematic illustration of the proton depletion process at a duty cycle of 0.6, which results in a pH higher than the baseline in the cathode compartment.

### 3.4 Voltammogram Shift

The RBIP’s effect on the electrochemical system was measured by comparing cyclic voltammograms of the Pt working electrode with the RBIP driven with various duty cycles. The cell was filled with a 2.6 mM of HCl aqueous solution. The sample was fabricated as described in the experimental section with an alumina ALD coating. The scan rate was 50 mV s^–1^. First, a voltammogram was taken with the RBIP OFF (*V*_in_ = 0 V). Then, a CV measurement was carried out with a square wave input signal applied to the RBIP. Last, the input was set again to 0 V, and CV was remeasured. This procedure was repeated with the duty cycle varied between 0 and 1 in steps of 0.1. The frequency was 15 kHz, and the amplitude was *V*_p–p_ = 1.4 V. Figure 5a shows the measured voltammograms. The black dashed curves are voltammograms measured while the RBIP was OFF before and after each time the RBIP was ON, and the colored curves are voltammograms measured while various input signals were applied to the RBIP. The color coding corresponds to the input signal duty cycle. The RBIP operates as a voltage source, adding (or subtracting) to the potential applied by the potentiostat. As a result, the peaks and current onsets of the CV curves shift with the RBIP-induced voltage. For duty cycles below 0.5, the RBIP induced a cathodic voltage that drove protons toward the working electrode. As a result, the HER current onset was shifted anodically. However, for duty cycles above 0.5, RBIP drove protons away from the working electrode and the HER current onset shifted to more cathodic potentials. Conversely, the OER onset shifted cathodically for duty cycles above 0.5 and anodically for duty cycles below 0.5. Constant biasing of the RBIP (duty cycle of 0 and 1) did not affect the CV curve, as no voltage develops across the RBIP. The voltammograms with the ratchet OFF (*V*_in_ = 0 V) overlay almost perfectly, indicating minimal changes to the RBIP, solution, and electrodes during operation. We define the HER onset potential as the potential of the local cathodic current minimum (in terms of absolute value) just before the sharp increase in the cathodic HER current (note (i) in Figure 5a). The Pt working electrode proton desorption peak potential and current are the potential and current at which the curve reaches an anodic current maximum after driving the HER ((iii) in Figure 5a). Figure 5b shows the potentials of the HER onset and the proton desorption peak extracted from the CV curves in Figure 5a as a function of the input signal duty cycle. At duty cycles below 0.5, the voltage induced by the RBIP contributed to the HER. The proton desorption peak and HER onset potential reached potentials as high as 0.236 and 0.087 V vs RHE, respectively. At input signal duty cycles above 0.5, the RBIP induced a voltage that negates driving HER at the working electrode. As a result, the WE potential must be more cathodic to drive the same reaction, and the proton desorption peak and HER onset potentials reached −0.07 and −0.137 V vs RHE, respectively. The effect of the RBIP on the two potentials is very similar, indicating that in this configuration, the RBIP acts as a voltage source that is added to the potential applied by the potentiostat. This was verified by comparing the CV curves measured with the ratchet ON to CV curves measured in a potential range that is shifted by a magnitude similar to the voltage induced by the RBIP (see the Supporting Information section 2 for more details). Figure 5c shows the ratchet-induced current extracted from points (ii) and (iii) on the CV as a function of the input signal duty cycle (Figure S6 shows how the proton desorption peak ratchet-induced current is extracted). The voltage induced by the ratchet led to a shift in the HER current onset and a corresponding change in the HER and proton desorption currents. The resulting HER current change reached 9 and 8.7 μA in absolute value for duty cycles of 0.4 and 0.6, respectively. The proton desorption current change reached 4.9 μA (in absolute value) for a duty cycle of 0.4 and 5.4 μA for a duty cycle of 0.6.

**Figure 5:**
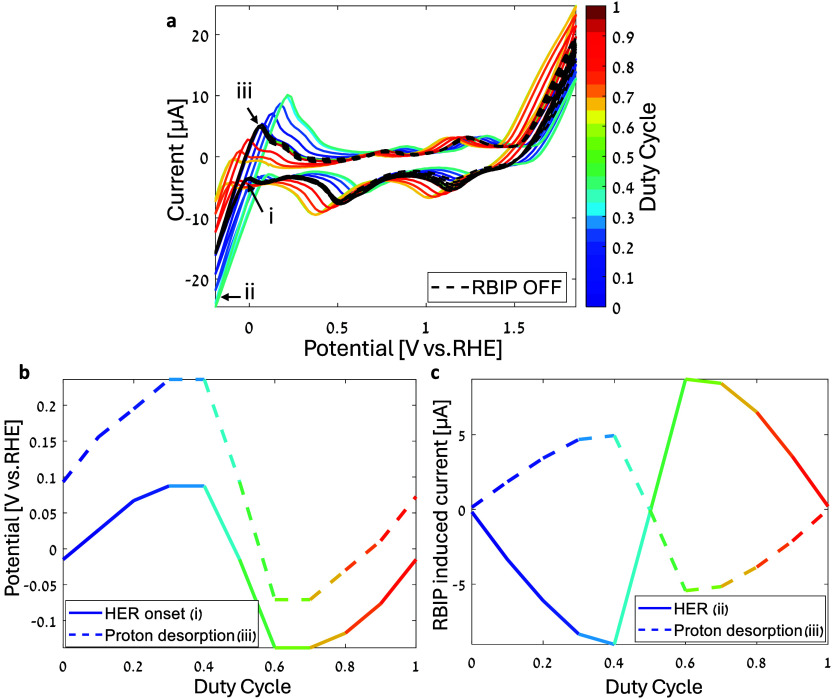
(a) Pt working electrode CV measurements with the RBIP OFF (*V*_in_ = 0, dashed black curves) and with the RBIP driven with several input signal duty cycles. The scan rate is 50 mV s^–1^. The sample was fabricated as described in the experimental section with an alumina ALD coating. The electrolyte is a 2.75 mM HCl aqueous solution. The input signal frequency is 15 kHz, and the amplitude is *V*_p–p_ = 1.4 V. (b) Potential of the HER onset and proton desorption peak extracted from (a) as a function of the input signal duty cycle. (c) Extracted RBIP-induced HER and proton desorption currents as a function of the input signal duty cycle.

The RBIP’s effect on the CV was also measured and analyzed in configuration B (Figure S3). For duty cycles below 0.5, the CV curves shifted anodically in configuration A (where the working electrode is in the *R* ^–^ compartment) and shifted cathodically in configuration B (in which the working electrode is in the *R* ^+^ compartment). Conversely, for duty cycles above 0.5, the CV curves shifted cathodically in configuration A, but shifted anodically in configuration B. This demonstrates that this sample induces a voltage that directs protons to the *R* ^–^ compartment when driven with a duty cycle below 0.5 and to the *R* ^+^ compartment with a duty cycle above 0.5. This experiment was repeated in a 1.6 mM H_2_SO_4_ aqueous solution and showed the same trends with slightly higher output (an anodic shift of 175.9 mV). Thus, the effects observed are a result of an inherent RBIP functionality and are not specific to the solution chemistry (see the Supporting Information section 4 for more information). To ensure that the observed ratchet action is not due to feedback introduced by the potentiostat, experiments were also conducted in a two-electrode arrangement. As with Figure 5a, the introduction of an alternating signal to the RBIP resulted in cathodic or anodic shifts in the current onsets and peaks according to the input signal duty cycle. However, the application of a constant bias to the RBIP had no effect on the CV. More information on these measurements can be found in the Supporting Information section 5.

RBIPs can be utilized as additional voltage sources that tune overpotentials (Figures 5 and S2) or as ion pumps (Figure 4). Using RBIPs as voltage sources to reduce overpotentials in fuel generating electrochemical systems may not lead to significant energetic gains. However, the effect of the RBIP on the current–voltage relationship can be viewed as that of a transistor where the voltage applied to one electrode (the gate) controls the current–voltage relationship between two other electrodes (source and drain). Thus, controlling the current between the working and counter electrodes by applying various signals to the RBIP can be utilized to obtain a transistor-like functionality. Combining this functionality with ion–ion selectivity^19^ can lead to the development of highly controlled electrochemical systems where only specific reactants are allowed to reach the electrodes. Such functionality may be useful in applications such as precise drug delivery systems and amplified ion-specific chemical sensors.

RBIPs may be most applicable for catalysis applications when they are used as ion pumps. For instance, pulsed electrochemistry has been shown to enhance reaction rates and modulate the selectivity of multiproduct electrochemical processes.^33,34^ Combining ratchet driven ion pumping with pulsed electrochemistry can result in synergy between these concepts. For example, by selectively pumping ions or intermediates toward an electrode at a frequency that enhances the formation of specific products. Catalytic resonances^35,36^ may be demonstrated by positioning the working electrode within a Debye length from the RBIP, allowing dynamic ion fluxes to directly influence surface reactions. Alternatively, one of the RBIP surfaces can be operated as a working electrode by alternating its potential between values that introduce catalytic resonances. Furthermore, applying the theoretical framework developed for selective ion pumping to the study of catalytic resonances may offer new insights into the role of the dynamics of the intermediates in the resonant catalytic process.

## 4 Conclusions

RBIPs were utilized as active membranes in an electrolysis cell. The application of an alternating input signal to the RBIP results in a buildup of ratchet-induced voltage between the two sides of the membrane. This voltage is then utilized to accelerate or suppress electrochemical reactions on the surface of the working electrode according to the input signal duty cycle. pH regulation was demonstrated by pumping protons toward the water-splitting cathode, thus compensating for proton depletion by the reaction. Conversely, at a higher input signal duty cycle, proton depletion was enhanced by pumping protons away from the cathode during the water-splitting process. CV measurements during the RBIP operation showed that the RBIP can shift the onset potential of electrochemical reactions by up to 142 mV, demonstrating an electrochemical transistor-like behavior. The RBIP’s ability to tune the onset potential of redox reactions and to regulate the chemical environment near electrodes can provide an added degree of freedom in electrochemical systems used for renewable fuels, chemical sensors, and other applications.

## Supplementary Material



## Notes

Experimental details; pH regulation in configurations A and B; CV shift; CV in configuration B; operation in sulfuric acid; two-electrodes experiment (PDF)
