# Supplementary material for: Tuning Electrochemical Reactions with Ratchet-Based Ion Pumps
Source: ACS Appl Energy Mater. 2025 Oct 4;8(20):15326–33. doi: 10.1021/acsaem.5c02349 (PMC12569968; doi:10.1021/acsaem.5c02349)
Supplement: Supplementary file 1 [file ae5c02349_si_001.pdf]

Supporting information
Tuning electrochemical reactions with ratchet-based ion pumps

Dafna Amichay,[†] Alon Herman,[†] Keren Shushan Alshochat,[†] Eden Grossman,[†] Baruch Hirsch,[†] Anchal Vashishtha,[‡,¶] Eran Edri,[‡,¶] Brian A. Rosen,[§] and Gideon Segev[*,†]

†*School of Electrical and Computer Engineering, Tel Aviv University, Tel Aviv 6139001, Israel.*

‡*Department of Chemical Engineering, Ben-Gurion University of the Negev, Be'er-Sheva 8410501, Israel.*

¶*Ilse Katz Institute for Nanoscale Science and Technology, Be'er-Sheva 8410501, Israel.*

§*Department of Materials Science and Engineering, Tel Aviv University, Tel Aviv 6139001, Israel.*

E-mail: gideons1@tauex.tau.ac.il

1 pH regulation in configurations A and B

The experiment described in Figure 4 was repeated in configurations A and B with a different sample. Figure S1a shows the pH measured in the two electrode compartments while the RBIP was disconnected and when it was operating. Figure S1b,c shows the ratchet-induced voltage measured in a Chronopotentiometry duty cycle sweep for configurations A and B, respectively. In all these measurements, the working electrode current is $-3\mu A$, the

compartments were filled with a 0.2mM HCl aqueous solution, the input signal frequency is $100Hz$, and the amplitude is $V_{p-p} = 1.4V$. The sample was fabricated as the one as in Figure 4. As shown in Figure S1b,c, the RBIP-induced voltage shows a typical ratchet behavior with low outputs at extreme duty cycles, and peaking at d_C =0.5 for both configurations. The uncertainty of the RBIP induced voltage is calculated as in the experimental section. In configuration A, the RBIP-induced voltage is negative for all duty cycles, indicating that a higher overpotential is required in order to maintain the same HER current. Thus, the RBIP is pumping ions away from the cathode compartment at every duty cycle. In configuration B, the RBIP output is positive. Thus, a lower overpotential is required in order to maintain the reaction. Hence, in this case, the RBIP pumps ions towards the working electrode for all duty cycles. As observed in Figure S1a, at a duty cycle of 0.7, when the system was in configuration A, protons were pumped away from the cathode compartment, and the pH rose above the baseline. In contrast, when the system was in configuration B (switching the position of the working electrodes), protons were pumped toward the cathode compartment, maintaining the pH closer to its initial value.

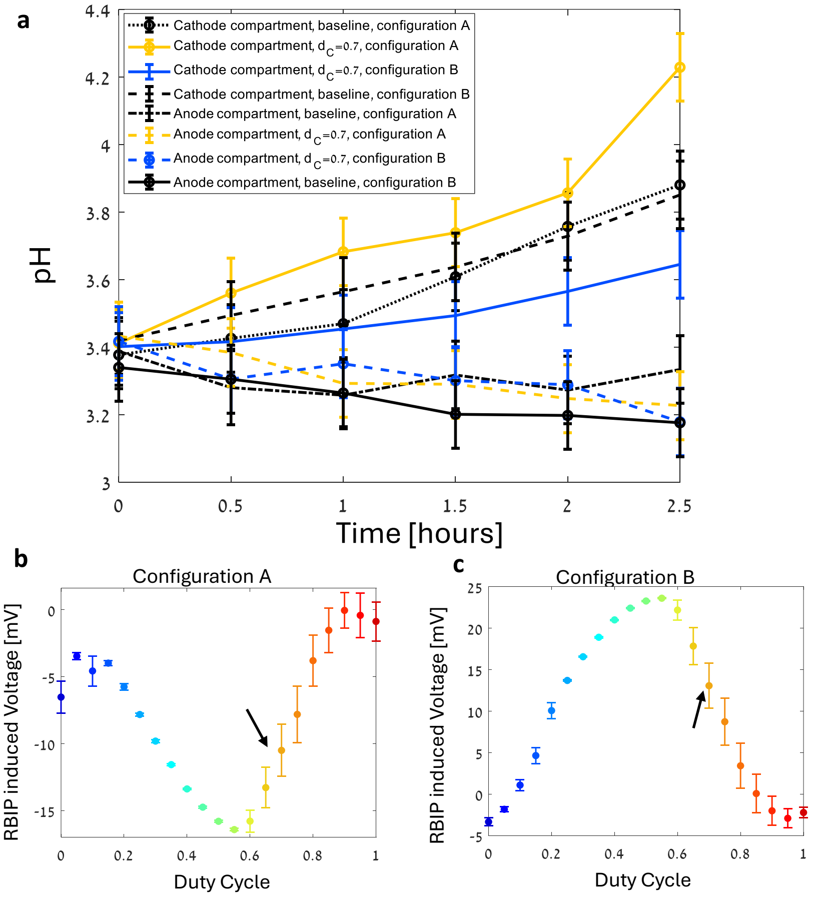

Figure S1: (a) The measured pH in the anode and cathode compartments when driving water splitting in configurations A and B, with the RBIP operating and when the RBIP was off ($V_{in} = 0$ V). (b-c) The ratchet-induced voltage measured in a Chronopotentiometry duty cycle sweep for configurations A and B, respectively. The working electrode current is $-3\mu A$, the compartments were filled with a 0.2mM HCl aqueous solution, the input signal frequency was $100Hz$, and the amplitude is $V_{p-p} = 1.4V$. The sample was fabricated as described in the experimental section with a TiO_2 ALD coating. The error bars in (b,c) mark the uncertainty as calculated using equation 5.

2 Cyclic voltammetry shift

The RBIP functions as a voltage source connected in series to the potentiostat, thus adding or subtracting from the voltage applied to the working electrode during the CV measurement. This was verified by comparing CV curves measured with the RBIP ON with CV curves measured with RBIP OFF, yet at a potential range that is shifted by the RBIP-induced voltage. First, CV curves were measured in the potential range between -0.342 V and 1.845 V vs RHE with the RBIP OFF and with the RBIP driven at duty cycles of 0.2 and 0.8. The frequency is 23 kHz, the electrolyte was 2.75 mM HCl aqueous solution, and all other parameters are as in Figure 5. Figure S2a shows the measured curves. When driven at a duty cycle of 0.2, the HER onset potential was shifted by 44 mV with respect to the HER onset when the ratchet is OFF. Similarly, for d_C =0.8 HER onset was shifted by -65.9 mV with respect to the onset potential when the ratchet was OFF. Next, two more CV measurements were taken with the RBIP OFF. Here the potential range was modified according to the voltage shifts found above: the first was measured between potentials of -0.386 V and 1.801 V vs. RHE (a 44 mV cathodic change in range with respect to the original potential range), and the second was measured between potentials of -0.276 V and 1.911 V vs. RHE (a 65.9 mV anodic change in range with respect to the original range). Figure S2b shows the measured curves and the curve measured initially with the ratchet OFF. Last, each of the curves in Figure S2b was compared to the curve measured with the RBIP ON with the duty cycle that induced the corresponding potential shift. Figure S2c, shows the CVs measured with RBIP driven with a duty cycle of 0.2 (solid line) and the CV measured with the RBIP OFF and the modified potential range (dashed line). To better compare the shapes of the two curves, the latter CV was artificially shifted anodically by 44 mV by adding the potential shift to its potential data. Similarly, the CV measured with the RBIP driven at a duty cycle of 0.8 was compared to the CV measured with the RBIP OFF, and the CV potential range was augmented by -65.9 mV. Here, the CV curve measured with the RBIP OFF was artificially shifted cathodically by 65.9 mV by subtracting 65.9 mV from

its potential data. The comparison between these curves is shown in Figure S2d. The curves with the RBIP ON and the curves with the RBIP OFF and modified potential range show excellent agreement. Specifically, the OER and HER current onsets match almost perfectly (Figure S2c,d). This demonstrates that in this electrode configuration, the RBIP functions as an additional voltage source, similar to an added potential induced by the potentiostat. If the RBIP functioned as a variable ionic resistance, it would have caused the slopes of the CV curve to change above the current onset and would not have shifted the curves as observed.

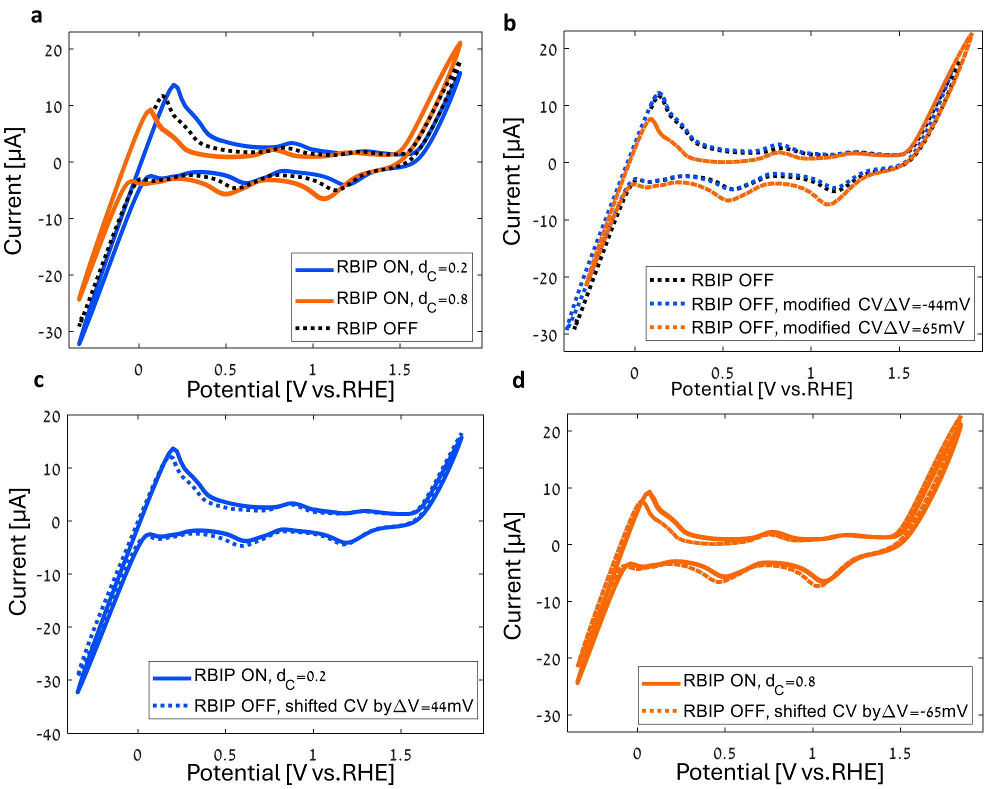

Figure S2: (a) three electrode cyclic voltammetry measurement using a scan rate of $50mVs^{-1}$ when the RBIP is off and when the RBIP is ON with d_C =0.2 and d_C =0.8. The frequency is 23 KHz and the amplitude is $V_{p-p} = 1.4V$. The RBIP was fabricated as described in the Experimental section with an alumina ALD coating. (b) CV curves measured with the RBIP OFF and the potential range modified by the potential shifts induced by the RBIP in (a). (c) A comparison between the CV curve measured in (a) for a duty cycle of 0.2 and the CV from (b) with the potential range modified by 44 mV. To better compare the shapes of the curves, the curve from (b) was artificially shifted cathodically by 44 mV by reducing 44 mV from its potential data. (d) A comparison between the CV curve measured in (a) for a duty cycle of 0.8 and the CV from (b) with the potential range modified by -65.9 mV. Here, the curve from (b) was artificially shifted anodically by 65.9 mV by adding 65.9 mV from its potential data.

3 Cyclic voltammetry in configuration B

The measurements depicted in Figure 5 were repeated with the electrodes in configuration B. Figure S3 shows a comparison between the CV curves measured in configurations A and B for various input signal duty cycles. Figure S3a shows the same data as in Figure 5a (configuration A), and Figure S3d shows the corresponding CV curves measured in configuration B. Figure S3b,e shows respectively the RBIP induced currents in configurations A and B, and Figure S3c,f shows respectively the HER onset and proton desorption potentials in configurations A and B. When switching the electrode configuration, the shift in overpotential and current is reversed, revealing the ratchet directionality. In configuration B, for duty cycles below 0.5, the HER onset and proton desorption peak are shifted to more cathodic potentials (Figure S3f), leading to a current decrease (Figure S3e). At duty cycles above 0.5, the HER onset and proton desorption peak are shifted to more anodic potentials, resulting in an increase in current. Thus, as shown in Figure 3, the RBIP drives protons towards the R^- compartment for duty cycles below 0.5 and towards the R^+ compartment for duty cycles above 0.5.

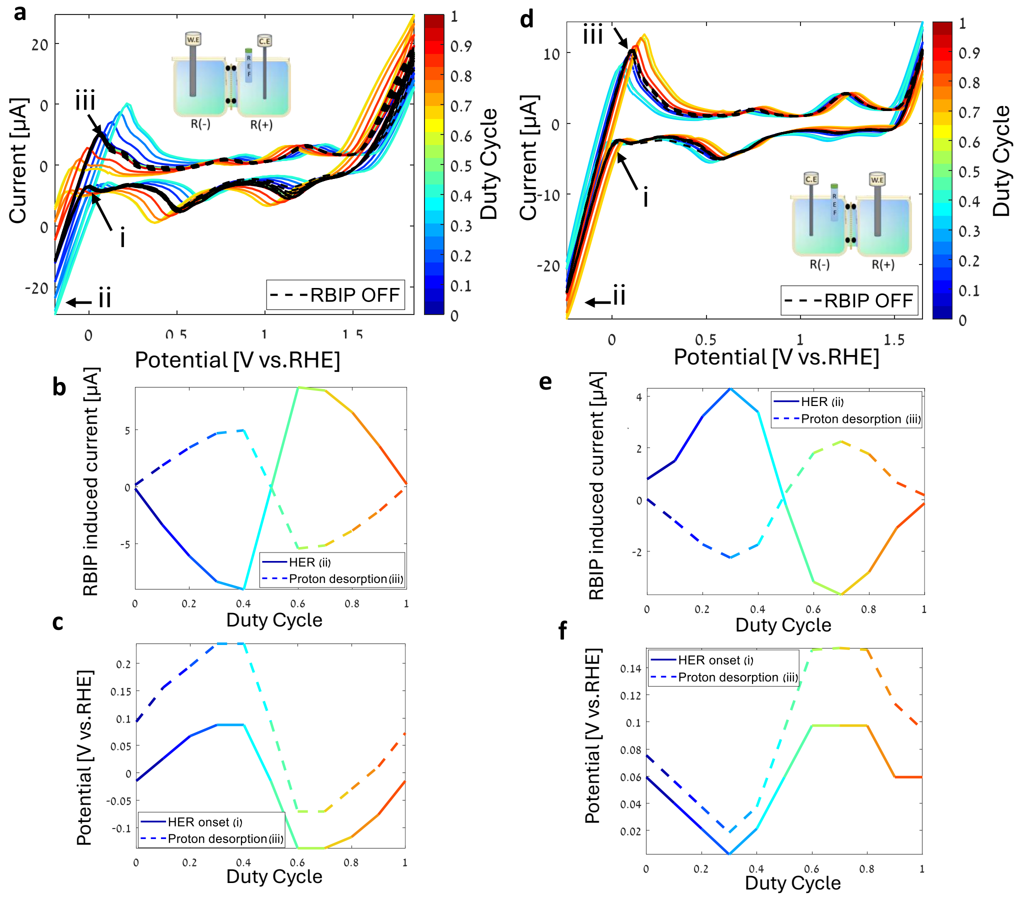

Figure S3: (a,d) Three-electrode cyclic voltammetry curves measured in configurations A and B respectively, with the RBIP OFF (V_{in}= 0 V, dashed black curve) and with the RBIP driven at several input signal duty cycles. The scan rate is $50mVs^{-1}$. The RBIP was fabricated as described in the Experimental section with an alumina ALD coating. The electrolyte is a 2.75 mM HCl aqueous solution. The input signal frequency is 15 kHz, and the amplitude is $V_{p-p} = 1.4V$. (b) The RBIP induced current as a function of the duty cycle in configuration A extracted from (a). (c) The HER onset and proton desorption peak potentials as a function of the input signal duty cycle in configuration A as extracted from (a). (e) The RBIP induced current as a function of the duty cycle in configuration B as extracted from (d). (f) The HER onset and proton desorption peak potentials as a function of the input signal duty cycle in configuration B as extracted from (d).

4 Operation in sulfuric acid

The effect of the RBIP on the CV curves was also studied in sulfuric acid. The measurement procedure was as in Figure 5. Figure S4a shows the CV curves measured with the RBIP OFF ($V_{in} = 0V$, dashed black curve) and with input signals with various duty cycles applied to the RBIP. The scan rate is $50mVs^{-1}$ and the electrodes are in configuration A. The RBIP sample fabrication process was the same as used for the sample discussed in Figure 5, and the electrolyte is a 1.6 mM H_2SO_4 aqueous solution. The input signal frequency is 23 kHz, and the amplitude is $V_{p-p} = 1.4V$. Figure S4b shows the HER onset and the proton desorption potentials as a function of the input signal duty cycle, and Figure S4c shows the RBIP-induced HER and proton desorption current change as a function of the input signal duty cycle. The CV curves show the same trends as the results in HCl aqueous solution. The output is slightly higher than measured in HCl reaching a maximal HER current change of $-11.42\mu A$ and $11.62\mu A$ for duty cycles of 0.4 and 0.6, respectively, and an HER onset potential shift of $175.9mV$ and $-153mV$ for duty cycles of 0.4 and 0.6, respectively.

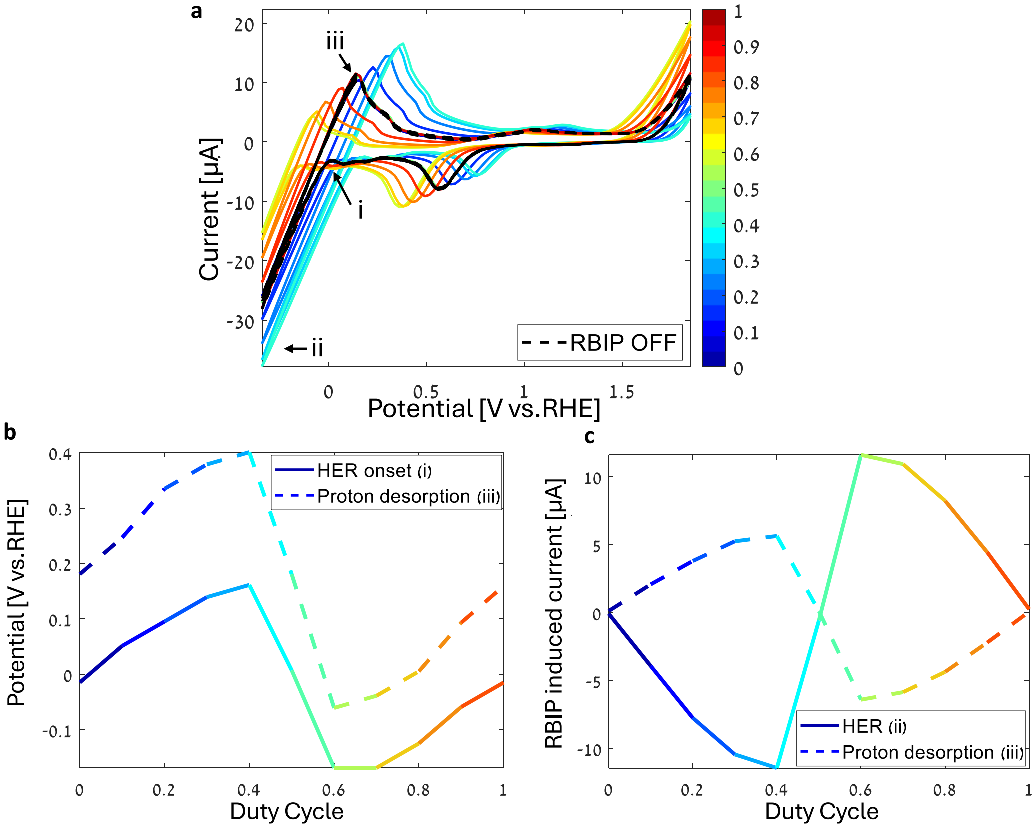

Figure S4: (a) Three-electrode cyclic voltammetry measurements when the RBIP is OFF (V_{in} = 0 V, dashed black curves) and with the RBIP driven with several input signal duty cycles. The scan rate is $50 mV s^{-1}$. The RBIP was fabricated as described in the Experimental section with an alumina ALD coating. The electrolyte was a 1.6 mM H_2SO_4 aqueous solution. The input signal frequency was 23 KHz, and the amplitude was $V_{p-p} = 1.4V$. (b) The extracted HER onset and proton desorption potentials as a function of the input signal duty cycle. (c) The extracted RBIP-induced HER and proton desorption current change as a function of the input signal duty cycle.

5 Two-electrodes experiment

To assure that the ion pumping effect is not an artifact introduced by electronic feedbacks in the potentiostat, the CV measurements were also conducted in a 2-electrode configuration. The CV curve was measured once when the ratchet was OFF, and then was measured with the ratchet ON at duty cycles of 0.2 and 0.8. The input signal frequency was 10 kHz, and the amplitude was $V_{p-p} = 1.4V$. The electrolyte is a 1.6 mM H_2SO_4 aqueous solution. Last, the CV curve was measured while a constant bias of -420 mV (the time-averaged voltage for an input signal with a duty cycle of 0.2) was applied to the RBIP. The solution and sample were as in section 4 in the supplementary information. Figure S5 shows the measured CV curves. The trends are similar to the results observed in the 3-electrode experiments: when a square wave is applied, the CV shifts in potential according to the input signal duty cycle. However, a constant bias has no effect on the CV.

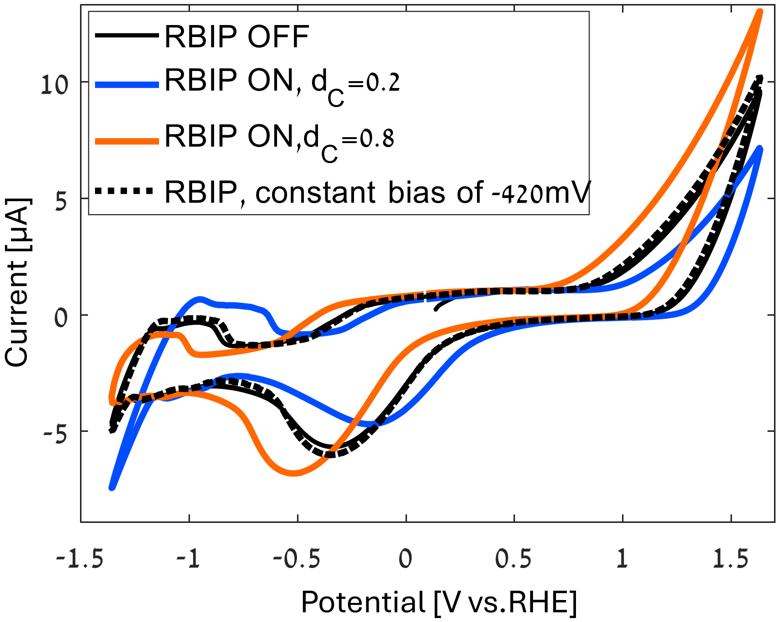

Figure S5: Two-electrode cyclic voltammetry curves measured with the RBIP OFF ($V_{in} = 0V$), driven with duty cycles of 0.2 and 0.8, and when a constant bias of -420 mV was applied to the RBIP. The input signal frequency was 10 kHz, and the amplitude was $V_{p-p} = 1.4V$. The scan rate is $50mVs^{-1}$. The electrolyte is a 1.6 mM H_2SO_4 aqueous solution. The RBIP was fabricated as described in the Experimental section with an alumina ALD coating.

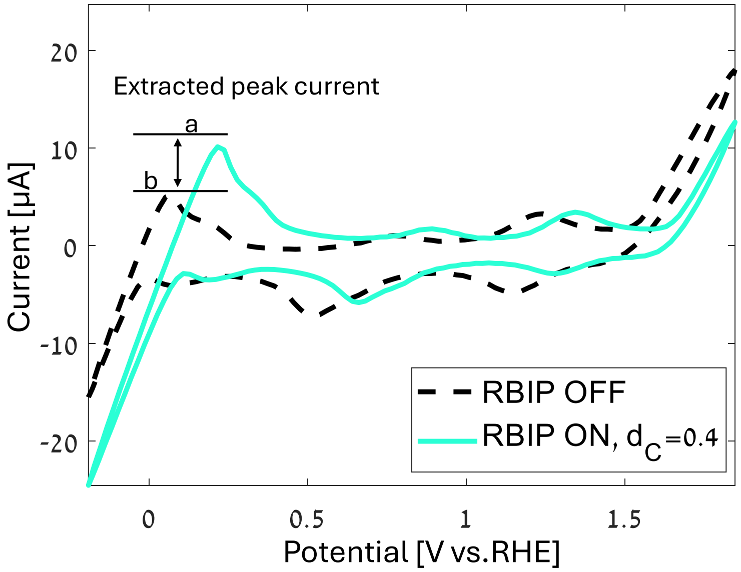

Figure S6: graphical calculation example for the extracted RBIP induced current as discussed in Figure 5
